# Inspiration After Posterior Pharyngeal Flap Palatoplasty: A Preliminary Study Using Computational Fluid Dynamic Analysis

**DOI:** 10.3389/fped.2022.823777

**Published:** 2022-05-03

**Authors:** Chao Yang, Jiang Li, Huo Li, Nan Chen, Xing Yin, Bing Shi, Jingtao Li, Hanyao Huang

**Affiliations:** ^1^Department of Oral Maxillofacial Surgery, State Key Laboratory of Oral Diseases, National Clinical Research Center for Oral Diseases, West China Hospital of Stomatology, Sichuan University, Chengdu, China; ^2^The Third People’s Hospital of Chengdu, Clinical College of Southwest Jiaotong University, The Second Affiliated Chengdu Hospital, Chongqing Medical University, Chengdu, China; ^3^Department of Pharmacy, Personalized Drug Therapy Key Laboratory, Sichuan Province Sichuan Academy, Medical Sciences and Sichuan Provincial People’s Hospital, School of Medicine, University of Electronic Science and Technology of China, Chengdu, China; ^4^Department of Epidemiology and Health Statistics, West China School of Public Health and West China Fourth Hospital, Sichuan University, Chengdu, China; ^5^Department of Orthodontics, State Key Laboratory of Oral Diseases, National Clinical Research Center for Oral Diseases, West China Hospital of Stomatology, Sichuan University, Chengdu, China

**Keywords:** cleft palate, velopharyngeal closure, posterior pharyngeal flap, computational fluid dynamics, palatoplasty complications

## Abstract

Posterior pharyngeal flap palatoplasty (PPF) is one of the most commonly used surgical procedures to correct speech, especially for patients suffering from velopharyngeal insufficiency (VPI). During PPF, surgeons use the catheter to control the lateral velopharyngeal port on each side. Airway obstruction and sleep apnea are common after PPF. To understand the air dynamics of the upper airway after PPF, we used computational fluid dynamics (CFD) to demonstrate the airflow. In our previous study, we have revealed the expiration process of the upper airway after PPF and shown the features of how PPF successfully restores the oral pressure for speech. In this study, we focus on examining the inspiration process. Normal airway structures were included. For the normal velopharyngeal structure, one cylinder was applied to each model. For recapitulating the velopharyngeal structure after PPF, two cylinders were used in each model. The ports for borderline/inadequate closure, which can help the oral cavity get the required pressure, were chosen for this study. A real-time CFD simulation was used to capture the airflow through the ports. We found that the airflow dynamics of the upper airway’s inspiration were dependent on the velopharyngeal structure. Although the airflow patterns were similar, the velocities between one-port and two-port structures were different, which explained why patients after PPF breathed harder than before and suggested that the one-port structure might be a better choice for secondary VPI reconstruction based on the CFD analyses.

## Introduction

A typical speech requires the soft palate to rise and make contact with the posterior pharyngeal wall to close off the nasal cavity from the mouth. Velopharyngeal insufficiency (VPI) happens when this contact is loose, or sometimes no contact exists. Under these circumstances, air can leak into the nose, causing hypernasal vocal resonance and nasal emissions (1). Although there is no gold standard for surgical repair of VPI, posterior pharyngeal flap palatoplasty (PPF) is one of the most commonly used surgical procedures to correct speech, especially for patients suffering from VPI (2–4). The superiorly based pharyngeal flaps were the most commonly performed procedure, ideal for patients with good lateral pharyngeal wall movement but poor palate movement (5).

During PPF, surgical adhesion of the soft palate to the posterior pharyngeal wall is performed, and a 4-mm-diameter catheter is recommended for controlling the velopharyngeal port on each side (6–8). Although PPF is a reliable surgical maneuver for palatal reconstruction, a few unavoidable complications are bothering patients postoperatively, including airway obstruction and sleep apnea. As a result, patients may need further surgery to adjust the ports to correct these problems (9). A 4-mm-diameter catheter represents an approximate 12.5 mm^2^ area, and according to its flexibility, it can help control the ports up to 10 mm^2^. The concept of controlling the size of the port in 10 mm^2^ was based on Warren’s pressure-flow device outcomes, which demonstrated that inadequate closure happened when the velopharyngeal port area was more extensive than 20 mm^2^ (10–12). Because the one-port structure was changed to the two-port structure, the final size of the port area, which is 10 mm^2^, was just divided from 20 mm^2^. However, based on recent computational fluid dynamic (CFD) analyses for velopharyngeal conditions, the airflow dynamics were much more complicated (13–17). The velopharyngeal ports’ size was calculated to be more than 13.34 mm^2^ when inadequate closure occurred, and different velopharyngeal closure patterns led to different airflow dynamics (13, 15). Meanwhile, the reasons for airway complications have been related to tonsils, high flaps, vertical advancement donor site closure method, and velocardiofacial syndrome (18). How the airflow is changed and the comparison of the airflow before and after PPF has never been revealed. Understanding the changes can help avoid airway complications and improve VPI care.

Our previous study has demonstrated the process of airflow before the speech (airflow from the lung to the oral cavity) in the upper airway after PPF (14). In this study, to understand the inspiratory process’s airflow after PPF, we again applied real-time CFD to demonstrate the upper airway’s air velocity and pressure. The models for normal velopharyngeal closure and velopharyngeal closure after PPF were shown before (14). The velopharyngeal ports were replaced by one cylinder and two cylinders, and inspirations with different velopharyngeal ports were recapitulated. We tried to find the differences in the airflow between the two kinds of structures.

## Materials and Methods

### Study Individuals and Airway Reconstruction

**FIGURE 1:**
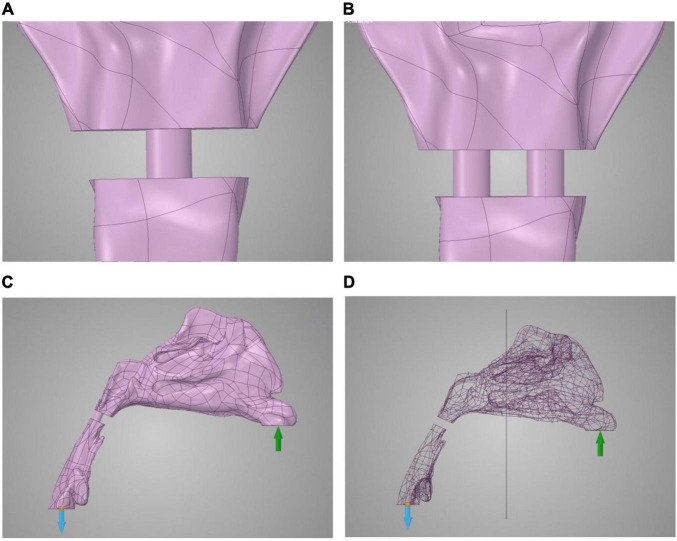
Model reconstruction of normal velopharyngeal closure and velopharyngeal closure after PPF. **(A)** One cylinder for one port under normal velopharyngeal closure, **(B)** two cylinders for the two ports under velopharyngeal closure after posterior pharyngeal flap palatoplasty, **(C)** the boundary set, and **(D)** the plane to divide the whole airway for calculating the airflow pressure in the nasal cavity. The green arrow represents the input of airflow, and blue arrow represents the output of airflow.

Study individuals and airway model reconstructions were described in our previously published study (14). The normal airway structures of seven individuals (three men and four women, age, 20–31 years), with no notable abnormalities (such as sleep-related symptoms or sleep apnea), were included. For the normal velopharyngeal structure, one cylinder (radius, 2.82 mm; height, 4.5 mm) was applied to each model (Figure 1A). Two cylinders (radius, 2.00 mm; height, 4.5 mm) were applied to each model to recapitulate the velopharyngeal structure after posterior pharyngeal flap palatoplasty (Figure 1B). Using the cylinder as the replacement allows us to control the variables by changing its radius. ANSYS Discovery Live (DL) (ANSYS Inc., Canonsburg, PA, United States) was used for model manipulation under both circumstances. The cylinders’ inferior areas were perpendicular to the trachea’s posterior wall and crossed the anterior edge of the atlas. Each cylinder was tangent to the airway’s posterior wall. The distance between the two cylinders was set at 4 mm based on the shape of the PPF. The ports for borderline/inadequate closure, which can help the oral cavity get the required pressure, were chosen (14).

### Airflow Dynamic Simulation of Inspiration

The real-time CFD simulation was performed under laminar, steady-state airflow at 35°C in the inspiration direction (19, 20). The nasal walls were non-slip and rigid. The gauge pressure was 0 Pa at the proximal end of the airway (Figure 1C). The inspiratory airflow of the inlet condition at the nostrils was 200 ml/s (21). The time of each process was set at 0.1 s for comparison. The fidelity of calculation was set to the maximum in the software. The airflow pressure and velocity of the whole airway and the ports, as well as the airflow pressure at the half-site of the entire airway (Figure 1D), were demonstrated by ANSYS Discovery Live (ANSYS Inc.). The scale bar cannot be fixed in this study because the real-time computational fluid dynamics were transient, and the scale bar was changing with time.

### Statistics

For testing the difference between PPF (two ports) and normal velopharyngeal closure (one port), the velocity and pressure at the orifice areas at the end of the calculation process (0.1s) were used. A paired *T*-test was applied to compare the velocity and pressure levels at the orifices between two manipulations of the same individuals. *P*-values <0.05 were considered to be significant.

The research protocol was censored and approved by the Ethics Committee of West China Hospital of Stomatology, Sichuan University (Approval No. WCHSIRB-D-2016-084R1). Individual participants could not be identified during or after data collection. Written informed consent was acquired from all the individuals enrolled in this study.

## Results

### Airflow Velocity Patterns Through the Upper Airways

**FIGURE 2:**
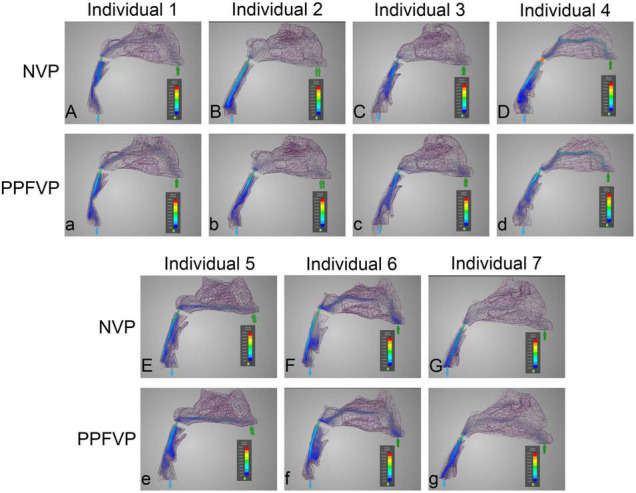
Airflow velocity patterns through the upper airways. The airflow velocity patterns of seven individuals were demonstrated. The color of the airflow was used to show the velocity changing in the same model. NVP, normal velopharyngeal closure (one port); PPFVP, velopharyngeal closure after posterior pharyngeal flap palatoplasty (two ports). The scale bar cannot be fixed because of the real-time simulation process in which the scale bar was changing with time.

Computational fluid dynamics demonstrated airflow velocity patterns. Figure 2 shows the airflow velocity through the upper airway. The inspiration process of the one-port and two-port velopharyngeal closures showed no significant difference according to the airflow velocity patterns in the same individual. All the individuals showed a slight velocity increase from the nasal vestibule to the region of three turbinates, and the velocity would decrease when getting into the turbinates. The velocity was lowest in the nasopharynx. The highest velocity happened at the velopharyngeal port.

Differences can be found between different individuals. In individuals 3 and 5 (Figures 2Cc,Ee), the main airflow was found to flow through the inferior turbinate. In the other five individuals (Figures 2Aa,Bb,Dd,Ff,Gg), the middle turbinate was the main airflow path.

### Airflow Pressure Patterns Through the Upper Airways

**FIGURE 3:**
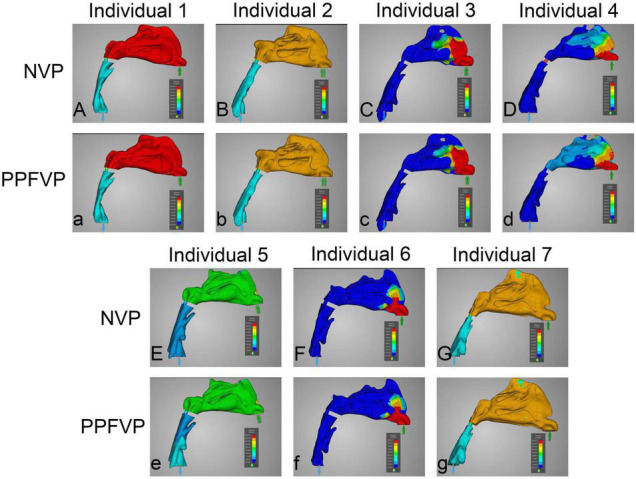
Airflow pressure patterns through the upper airways. The airflow pressure patterns of seven individuals were demonstrated. The color of the airflow was used to show the pressure changing in the same model. NVP, normal velopharyngeal closure (one port); PPFVP, velopharyngeal closure after posterior pharyngeal flap palatoplasty (two ports). The scale bar cannot be fixed because of the real-time simulation process in which the scale bar was changing with time.

**FIGURE 4:**
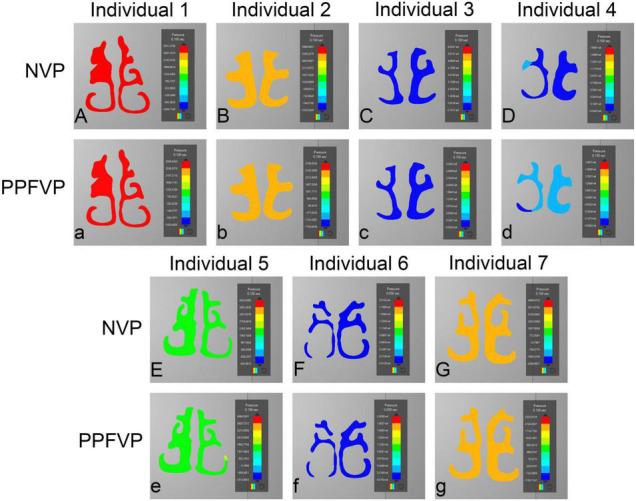
Airflow pressure patterns through the turbinates. The airflow pressure patterns through the turbinates of seven individuals were demonstrated. NVP, normal velopharyngeal closure (one port); PPFVP, velopharyngeal closure after posterior pharyngeal flap palatoplasty (two ports). The scale bar cannot be fixed because of the real-time simulation process in which the scale bar was changing with time.

Figures 3, 4 show the airflow pressure patterns of seven individuals. There was no significant difference between one-port and two-port structures in the same individual, except for individual 4. The nasal cavity pressure was significantly higher than the airway below the velopharyngeal port in individuals 1, 2, 5, and 7 (Figures 3Aa,Bb,Ee,Gg). In individuals 3 and 6, the highest pressure happened at the nasal vestibule to the turbinates. The difference between one-port and two-port structures was found in individual 4 (Figures 3Dd, 4Dd).

### The Difference in the Inspiration Process Between One-Port and Two-Port Structures

**FIGURE 5:**
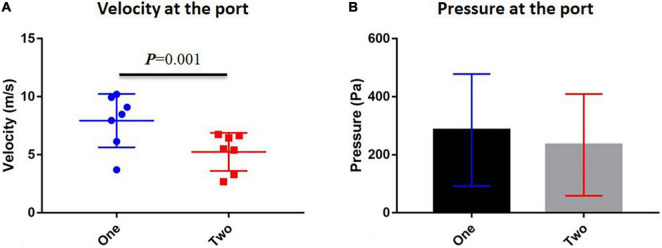
Comparison between one-port and two-port velopharyngeal closures. **(A)** Velocity at the velopharyngeal port and **(B)** pressure at the velopharyngeal port. Error bars are S.D.

Table 1 shows the velocity and pressure of each individual at the velopharyngeal port simulated by CFD. The paired *T*-test was used to compare the velocity and pressure at the orifice between one-port and two-port structures (Figure 5 and Supplementary Table 1). The velocity at the velopharyngeal port of the one port was significantly different from the two ports. The pressures at the velopharyngeal port were the same between the two situations.

**TABLE 1:** Velocity and pressure at the orifice of the VP port of each individual.

Individual	Normal VP closure (one port)		VP closure after PPF (two ports)	
	Velocity (m/s)	Pressure(Pa)	Velocity (m/s)	Pressure(Pa)
1	8.48	404.58	5.51	548.63
2	10.21	586.22	6.74	370.4
3	6.14	55.36	3.3	139.89
4	9.1	213.26	5.41	192.31
5	7.96	318.61	6.64	225.51
6	3.71	54.71	2.68	12.45
7	9.94	363.97	6.44	149.44

## Discussion

For decades, PPF palatoplasty has been applied to secondary VPI for restoring speech and correcting abnormal hypernasal vocal resonance and nasal emissions (1, 22). After PPF, the two-port fixed velopharyngeal structure replaces the normal one-port movable velopharyngeal structure. Before the surgery, the velopharyngeal port was too large to close or did not function normally to guarantee enough oral pressure for speech when VPI happened (23, 24). Thus, PPF helps narrow down the connecting tunnel between nasal and oral cavities and divides one large tunnel into two smaller tunnels. The oral pressure under the two-port circumstances could reach the required magnitude much more accessible than before (14). However, the weaknesses of this surgical method are apparent. Airway obstruction is one of the most significant problems caused by PPF, affecting the patients’ quality of life (9).

As imaging and computational tools have been improved over the past decades, CFD has become an efficient method for researching patient-specific airway models (25). It has been applied to study cleft palate-related airflow (13–15, 26). CFD can provide visible outcomes that demonstrate the real-time airflow and precisely calculate the fluid parameters. Those parameters can be compared between samples quickly. Thus, we again applied CFD to help us understand the fluid dynamic characteristics of the upper airways of patients with PPF.

The mechanism of PPF, which was to connect the posterior pharyngeal wall physically, was clear (1). The port area was the most important because it directly influences the surgical outcomes. It was also reported that the size of the pharyngeal flap (large, medium, and narrow) could be decided by preoperative velar and pharyngeal movement (27), which in turn could affect the postoperative outcomes. If the ports were too small, the patients would find it difficult to breathe with the nose as the VP ports cannot be changed (28). Then they prefer to breathe with their mouth. It could cause more problems such as abnormal maxillofacial growth (29). A large port might not guarantee appropriate oral pressure for speech. According to results from a pressure-flow measurement model by Dr. Warren’s team (10–12), the area of two ports should be fixed at 10 mm^2^ (8).

Our previous study has found that the area should be around 9.67 mm^2^, similar to Warren’s recommendation of 10 mm^2^ (14). However, the air dynamics were proven to be changed with the two ports when we demonstrated the speech process in the two-port velopharyngeal structure. The speech process was like an expiration process, and significant differences were revealed. The inspiration process should also be analyzed to comprehensively understand the air dynamic changes in the upper airway and those complications such as nasal obstruction. This study focused on this purpose and performed real-time CFD simulation to elucidate the inspiration process characteristics of the two-port velopharyngeal structure.

As we controlled the ports not based on the area but the velopharyngeal function status, the ports’ total areas were different under different manipulations of the airway model from the same patient sample. We had demonstrated the reasons in our study that the ports’ areas could not be simply divided as the whole airflow system had been changed after changing the structure (14). When we performed the PPF to correct secondary VPI, we hoped the surgery could help the patient’s velopharyngeal structure to function like ordinary people. Thus, studying and comparing the airflows of one-port and two-port structures when the velopharyngeal functions are the same should be the right clue.

Interestingly, there was no significant difference in the inspiration airflow patterns between one-port and two-port structures. The phenomena were explainable as we controlled the velopharyngeal closure condition. In this study, these two kinds of manipulations, such as one port and two ports, of the same model were used in the areas under which the airway model worked as a borderline/inadequate closure. The total areas were different between the two manipulations. In other words, the one-port and two-port velopharyngeal closures were in the same condition to gather enough oral pressure. The airflow through the same upper airway should show no significant difference regardless of the port’s shape or size when the functions were the same. It needed to be clarified and emphasized that under the same velopharyngeal closure condition as borderline/inadequate closure, the total areas of the ports of one-port and two-port velopharyngeal structures were different, which again proved that the dynamic airflow mechanism of the upper airway is complicated and the two ports cannot be divided from the one port.

The velocity at the orifice showed a significant difference between one-port and two-port groups. The velocities of the two-port structure were lower than those of the one-port structure. It could help us understand why patients suffered airway obstruction and had to use mouth breathing rather than nasal breathing (30). The volume of breathed air should be maintained at a stable magnitude, but under the two-port structure, the airflow velocity decreased, and patients needed more endeavor to get enough air for breathing. That also explained why patients would find it hard to breathe with their nose. Thus, it might suggest that surgeons used a surgical maneuver to remain only at one port and still give the patient the necessary oral pressure for speech. For example, the lateral pharyngeal flap would exclusively remain one port while narrowing down the velopharyngeal port (31). Our study suggested that the one-port structure might help patients feel better than the two-port structure while still guaranteeing the required oral pressure for speech. Anyway, this hypothesis needed further clinical studies, such as speech-based outcomes for the lateral pharyngeal flap, and specific analyses to confirm.

In summary, we first used the simplified models to define the airflow areas for different VP conditions, which built the foundation for the following several CFD analyses (13). We compared different VP closure patterns and found that the patterns could affect the VP conditions (15). Based on this study, it was suspected that many factors could influence airflow dynamics in the airway. PPF was one operation that would permanently change the airway structure, and because of the remained small ports, patients always felt it difficult to breathe with the nose. Thus, we again used the CFD to first check the VP conditions in different port areas with the two ports (14). In addition, in this study, to further show the whole process of the patients after PPF, the inspiration process was tested. Our study demonstrated the airflow of the inspiration comprehensively and supplemented our latest publication to show the whole speech process of patients after PPF palatoplasty. It could help the surgeon understand the PPF maneuver better and provide important physiological and clinical insights into the velopharyngeal port after PPF palatoplasty. With the help of CFD and our simplified models, we successfully demonstrated the cause of those problems in VPI patients.

Some shortcomings remained in our models. For example, due to the need for demonstrating how the changing of port areas affects the airflow and a lack of a more realistic port structure, the two ports were simplified as two cylinders. Due to the simplification, we did not select the patient samples with VPI, but the validation should be done in the future. This study had to follow our last publication about the expiration of PPF and applied the same manipulations to the models. Moreover, the inspiration rate could not stay constant under real circumstances, although this is negligible due to the recording’s short duration. The setting of acquiring the data at 0.1 s was used to compare the airflow differences between structures, which can be changed according to the purpose of the study and should be validated with clinical measurement for the time of getting enough oral pressure before the speech. The airway resistance should be analyzed, and the validation of this CFD methodology by comparing the model predictions with actual surgical outcomes should be completed in future studies. Different times of daily life also could affect patients’ inspiration, such as at rest or during exercise and sleeping or awake so that the inlet condition might change. ANSYS Discovery Live is a good example for clinical use, which shows directly to the patients how the airflow through their airway quickly; however, if increased accuracy and high-fidelity of the calculated outcomes such as pressure or velocity are required, CFD software based on finite elements is recommended.

## Conclusion

Airflow dynamics of inspiration in the upper airway were found to be dependent on the velopharyngeal structure. Although the airflow patterns were similar, the velocities between one-port and two-port structures were different, which explained why patients after PPF breathed harder than before and suggested the one-port structure might be a better choice for secondary VPI reconstruction.

## Data Availability Statement

The original contributions presented in the study are included in the article/Supplementary Material, further inquiries can be directed to the corresponding author.

## Ethics Statement

The studies involving human participants were reviewed and approved by the Ethic Committee of West China Hospital of Stomatology, Sichuan University. The patients/participants provided their written informed consent to participate in this study.

## Author Contributions

CY, JaL, and HL contributed equally to this study and analyzed the data. CY, JaL, HL, NC, and XY contributed to the collection of data. CY, JaL, HL, NC, XY, BS, and HH contributed to writing and revising this study. BS, JtL, and HH supervised the study. All authors contributed to the article and approved the submitted version.

## Conflict of Interest

The authors declare that the research was conducted in the absence of any commercial or financial relationships that could be construed as a potential conflict of interest.

## Publisher’s Note

All claims expressed in this article are solely those of the authors and do not necessarily represent those of their affiliated organizations, or those of the publisher, the editors and the reviewers. Any product that may be evaluated in this article, or claim that may be made by its manufacturer, is not guaranteed or endorsed by the publisher.
