# Supplementary material for: Inspiration After Posterior Pharyngeal Flap Palatoplasty: A Preliminary Study Using Computational Fluid Dynamic Analysis
Source: Front Pediatr. 2022 May 3;10:823777. doi: 10.3389/fped.2022.823777 (PMC9111012; doi:10.3389/fped.2022.823777)
Supplement: Supplementary file 1 [file Table_1.docx]

### Supplemental Table

**Supplemental Table 1. Paired T Test of Parameters at Orifice**

| Value | Normal VP closure (one port) | | VP closure after PPF (two ports) | | Paired difference | | t value | ***P*** value |
| --- | --- | --- | --- | --- | --- | --- | --- | --- |
|  | Mean | SD | Mean | SD | Mean | SD |  |  |
| Velocity | 7.934 | 2.307 | 5.246 | 1.636 | 2.689 | 1.080 | 6.587 | 0.001 |
| Pressure | 285.244 | 192.922 | 234.090 | 175.356 | 51.154 | 137.278 | 0.986 | 0.362 |
